# Force sensor in simulated skin and neural model mimic tactile SAI afferent spiking response to ramp and hold stimuli

**DOI:** 10.1186/1743-0003-9-45

**Published:** 2012-07-23

**Authors:** Elmer K Kim, Scott A Wellnitz, Sarah M Bourdon, Ellen A Lumpkin, Gregory J Gerling

**Affiliations:** 1 Department of Systems and Information Engineering, University of Virginia, Charlottesville, VA, 22904, USA; 2 Gladstone Institute of Neurological Disease, San Francisco, CA, 94158, USA; 3 Departments of Dermatology and Physiology & Cellular Biophysics, Columbia University College of Physicians & Surgeons, New York, NY, 10032, USA

**Keywords:** Tactile, Force sensor, Leaky integrate-and-fire, Mechanoreceptor, SAI, Neural, Prosthetic, Biomechanics, Electrophysiology, Skin, Elasticity

## Abstract

**Background:**

The next generation of prosthetic limbs will restore sensory feedback to the nervous system by mimicking how skin mechanoreceptors, innervated by afferents, produce trains of action potentials in response to compressive stimuli. Prior work has addressed building sensors within skin substitutes for robotics, modeling skin mechanics and neural dynamics of mechanotransduction, and predicting response timing of action potentials for vibration. The effort here is unique because it accounts for skin elasticity by measuring force within simulated skin, utilizes few free model parameters for parsimony, and separates parameter fitting and model validation. Additionally, the ramp-and-hold, sustained stimuli used in this work capture the essential features of the everyday task of contacting and holding an object.

**Methods:**

This systems integration effort computationally replicates the neural firing behavior for a slowly adapting type I (SAI) afferent in its temporally varying response to both intensity and rate of indentation force by combining a physical force sensor, housed in a skin-like substrate, with a mathematical model of neuronal spiking, the leaky integrate-and-fire. Comparison experiments were then conducted using ramp-and-hold stimuli on both the spiking-sensor model and mouse SAI afferents. The model parameters were iteratively fit against recorded SAI interspike intervals (ISI) before validating the model to assess its performance.

**Results:**

Model-predicted spike firing compares favorably with that observed for single SAI afferents. As indentation magnitude increases (1.2, 1.3, to 1.4 mm), mean ISI decreases from 98.81 ± 24.73, 54.52 ± 6.94, to 41.11 ± 6.11 ms. Moreover, as rate of ramp-up increases, ISI during ramp-up decreases from 21.85 ± 5.33, 19.98 ± 3.10, to 15.42 ± 2.41 ms. Considering first spikes, the predicted latencies exhibited a decreasing trend as stimulus rate increased, as is observed in afferent recordings. Finally, the SAI afferent’s characteristic response of producing irregular ISIs is shown to be controllable via manipulating the output filtering from the sensor or adding stochastic noise.

**Conclusions:**

This integrated engineering approach extends prior works focused upon neural dynamics and vibration. Future efforts will perfect measures of performance, such as first spike latency and irregular ISIs, and link the generation of characteristic features within trains of action potentials with current pulse waveforms that stimulate single action potentials at the peripheral afferent.

## Introduction

Our sense of touch helps us perform activities of daily living, such as grasping a glass, discerning the structure of a coin, and buttoning a shirt. Completing these tasks proves difficult for the 541,000 U.S. citizens living with upper limb loss [1]. In the near future, however, advanced prosthetics may help reconstitute their motor and sensory function [2-4]. It is likely that sensory connectivity to the nervous system, via either peripheral afferent or cortex, will originate from mechanical sensors within an elastic skin. One major hurdle lies in modulating the delivery of the appropriate signals to the afferent [5], specifically both the shape of current pulses to elicit action potentials and the features within trains of action potentials. Work herein is focused upon the latter, in particular, those essential features captured by the slowly adapting type I (SAI) afferent in its response to the everyday task of contacting and holding an object. The intent is to complement and extend prior work with vibratory stimuli [6].

Among mechanoreceptive afferent types, SAI afferents respond to sustained compression as well as movement. SAI afferents exhibit firing rates about 10 times greater during stimulus movement than sustained hold, whereas their firing rates during held stimuli increases linearly with indentation depth from 0.1 to 2.0 mm [7]. SAI afferents exhibit comparatively high variability in the time interval between spikes.

Although both skin mechanoreceptors and artificial pressure sensors respond to compression, their outputs are quite different. Mechanoreceptive afferents in the skin produce trains of discrete action potentials, or “spikes,” whose rates represent salient stimulus features such as magnitude or velocity. In contrast, artificial force sensors output a continuous analog signal, typically with changes in resistance or capacitance that represent intensity [8,9]. The rate of intensity change is decoded from signal slope, rather than spike frequency. Other sensor technologies, including optical fibers [10] and tracking [11], output similar analog signals. Another difference between biological and artificial sensors is that mechanoreceptors respond to stress and/or strain at the location of natural tactile end organs, which lie embedded within the skin. Mechanics models clearly indicate that the skin influences the propagation of forces from its surface to the end organs, shaping the resultant neural response [12-16].

Work herein seeks to address these gaps and contribute to the knowledge base by computationally replicating how the slowly adapting type I (SAI) mechanoreceptor in its skin environment converts ramp-and-hold, sustained stimuli into spike trains that capture the features of contacting and holding an object. These features include the SAI’s increased neural firing to changes in stimulus magnitude and rate of change, the SAI afferent’s continuous firing response to sustained stimuli and greater response to moving stimuli. To address these gaps, this systems integration effort builds upon research that includes models of membrane transduction and neural dynamics by integrating a physical force sensor-elastic substrate with a mathematical model of neuronal spiking to transform sensor output to spike trains that capture the SAI response’s characteristic features. Model predictions are compared to electrophysiological recordings from mouse SAI afferents for similar ramp-and-hold stimuli. The rigorous engineering technique of response surface methodology was used to optimize the model’s six free parameters by following a gradient method of steepest descent to match predicted spike response with those observed in the SAI afferent, before separately validating the model. In addition, the work begins to consider the SAI afferent’s variable interspike interval and first spike latency.

## Methods

### Spiking-sensor model: force sensor-elastic substrate

**Figure 1:**
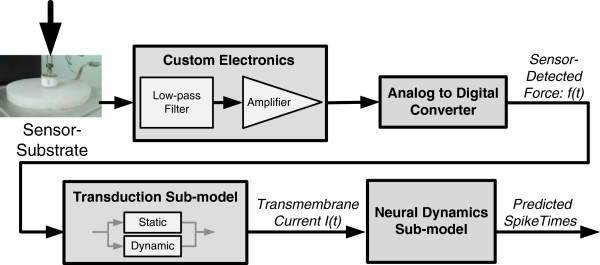
**Overview of system connectivity.** Sensor-substrate is connected to a filter, amplifier, and A/D converter to allow translation of sensor-detected forces to predicted spike times via the transduction and neural dynamics sub-models.

The first component of the spiking-sensor model was the physical force sensor-elastic substrate. A single piezo-resistive sensor (Flexiforce A201; Tekscan Inc., South Boston, MA) was embedded within a silicone-elastomer substrate (Figure 1). This sensor is commercially available, robust to damage when embedded within a silicone substrate, and integrated relatively easily with peripheral hardware and software. It responds to normal force within a range of 0 to 4.44 N over its thin (height = 0.20 mm) and circular (diameter = 9.53 mm) pressure sensitive area.

When applied load is increased over its sensitive area, the sensor's resistance decreases, allowing its conductance to increase linearly with respect to applied force. When integrated with custom-built circuitry, the voltage passed through the sensor is amplified and filtered (INA114; Burr-Brown Corporation, Tucson, AZ) before being fed to an analog-to-digital converter (DAQCard-6036E; National Instruments Corporation, Austin, TX), which collects data at a 100 Hz sampling rate. Supporting software (LabVIEW 8.5 Professional; National Instruments Corporation) was developed to record, calibrate, and translate analog voltage, *v(t)* in V, into force detected at the sensor's location, *f(t)* in N.

The voltage was linearly translated to force, per Tekscan's specifications and our own sensor calibration experiments. Noise from the sensor force output was filtered using a low-pass Gaussian filter to remove frequencies >15 Hz. For more information on the onset response of the Flexiforce sensor, see section *Discussion: Responsiveness of Sensor and Load Cell*.

The sensor was embedded within an elastic substrate to emulate the skin environment that typically surrounds mechanoreceptors. During the pouring and curing process of a cylindrical (diameter = 30 mm; height = 10 mm) silicone-elastomer substrate (TC5005 A/B-C; BJB Enterprises Inc., Tustin, CA), the sensor was embedded at a location in the center of the x–y plane and at a depth 1.0 mm below the surface. The specific depth of 1.0 mm, roughly emulating the depth of 0.5 to 1.0 mm of the SAI afferent’s Merkel cells in human skin [17], ensured that the embedded sensor responded over a maximal surface area (sensor diameter = 9.53 mm; surface receptive field diameter = 20 mm), while also minimizing its depth from the surface. Furthermore, the stiffness of the silicone-elastomer, controlled by varying the percentage of cross-linker (0.98%), was manipulated to match the modulus value (Young’s modulus = 136 kPa) reported for epidermal fingertip skin of human cadavers [15]. Although cadaver skin is stiffer than *in vivo* or *ex vivo* tissue, minimal empirical data on such stiffness exists. This modulus value is supported by finite element analysis [15,16,18,19].

### Spiking-sensor model: transduction sub-model

**Figure 2:**
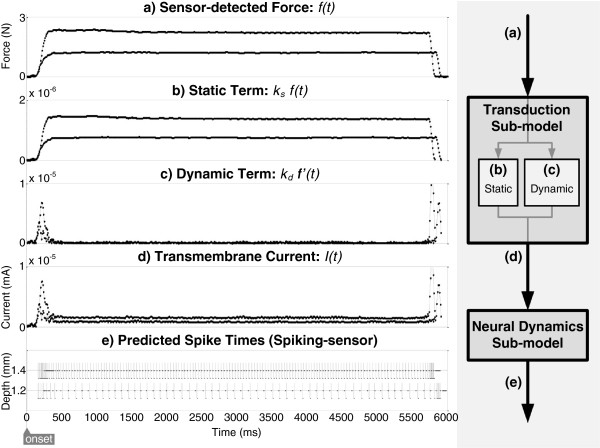
**Example transformations within the spiking-sensor model.(a)** Sensor-detected force is transformed (where components of **(b)** static magnitude and **(c)** dynamic change in force are summed) to create **(d)** current. The translated current predicts **(e)** spike times where membrane potential exceed threshold.

The second component of the spiking-sensor model was the mathematical transduction sub-model. Force detected at the sensor in the substrate (Figure 2a) was transformed into current (Figure 2d), similar to how stress and/or strain applied at an SAI afferent’s end organ is transformed into current across its membrane.

Unlike previous work by Lesniak and Gerling [20], which transformed strain energy density into transmembrane current using a sigmoidal function, the functions (1) and (2) developed here linearly convert sensor-detected force and change in detected force, *f’(t)* in N/ms, into current, *I(t)* in mA. Its three coefficient terms are the intercept constant *ß* in mA, the static gain *k*_*s*_ in mA/N, and the dynamic gain *k*_*d*_ in mA·s/N. The *ß* term is intended to account for the varying baseline between sensors. The change in detected force is calculated with a step-size resolution *h* of 10 ms, given 100 Hz sampling rate.

(1) $$I\left(t\right)=\beta +k_{s}f\left(t\right)+k_{d}f^{\prime }\left(t\right)$$

(2) $$f^{\prime }\left(t\right)=\{\begin{array}{c} 0\text{,}\;for\;t=onset\\ \left|\frac{f\left(t\right)-f\left(t-h\right)}{h}\right|\text{,}\;otherwise \end{array}$$

Although a sigmoid may more realistically reflect the behavior of sensory cells [21], the linear function minimizes the complexity of both the model and computational engine. Furthermore, the derivative represents the dynamic ramp-up phase of spike firing using only half the number of parameters required with a sigmoid.

The dynamic term, *k*_*d*_*f’(t)* as shown in Figure 1c, responds to a first-order change in sensor-detected force, and therefore dominates *I(t)* (Figure 2d) during both the ramp-up (<500 ms) and retraction portions of indentation, while the static term, *k*_*s*_*f(t)* as shown in Figure 1b, responds to the magnitude of force and contributes mainly during the sustained hold. Thus, the transduction sub-model accounts for stimulus adaptation. While the full-wave model suggests the retraction of the stimulus contributes to the vigorous elicitation of action potentials in this phase, a phenomenon exhibited in neural recordings [22,23], we did not perform an in-depth analysis here. The sub-model was implemented in C# and the values for parameters *ß*, *k*_*s*_, and *k*_*d*_ were determined through parameter fitting as described in section *Methods: Data analysis: parameter fitting*.

### Spiking-sensor model: neural dynamics sub-model

The third component of the spiking-sensor model was the mathematical neural dynamics sub-model. The transmembrane current was transformed into spike times (Figure 2e) by abstracting leaky integrate-and-fire behavior as a resistive-capacitive (RC) circuit [24,25]. First, current *I(t)* passes through a membrane with resistance *R* in Ohm and capacitance *C* in mF, to set the membrane potential *u(t)* in mV.

Once the membrane potential exceeds a threshold $\bar{v}$ in mV, an action potential is elicited. Upon firing, the time of the spike is recorded, the membrane potential is reset to rest, and the absolute refractory period is entered, during which no spike may be elicited. This entire process iterates until stimulus offset. While the biologically equivalent values of these parameters are known (i.e., typical resting membrane potential −65 mV), this sub-model simplifies resting membrane potential to 0 mV [26], so that relative to this baseline, the threshold is a positive value. The absolute refractory period was set to 1 ms [27,28].

This leaky integrate-and-fire behavior was mathematically defined by (3) and (4), where the membrane’s time constant τ, in units of ms, is the product of the membrane’s resistance and capacitance.

(3) $$I\left(t\right)=\frac{u\left(t\right)}{R}+C\frac{\partial u}{\partial t}\left(t\right)$$

(4) τ=RC

These two equations were combined and rewritten as a single differential equation

(5) $$g\left(t,u\right)=\frac{\partial u}{\partial t}\left(t\right)=\frac{-u\left(t\right)}{\tau }+\frac{I\left(t\right)}{C}\text{,}$$

where, *g(t,u),* the change in membrane potential is a function of time-dependent membrane potential, *u(t)*, and current, *I(t)*.

With the aid of a numerical, fourth-order Runge Kutta method for solving ordinary differential equations, the membrane potential in the next timestep *u*_*ti+1*_ – a linear combination of the differential equation evaluated at various points in time and membrane potential – is calculated iteratively until the future membrane potential *u*_*tN*_ reaches or surpasses the threshold. The resolution of simulated time increments was set to a constant step size of 0.01 ms. The initial membrane potential, *u*_*t0*_ which represents the baseline resting membrane potential, was set to zero.

The neural dynamics sub-model implemented in C# does not account for intrinsic neuronal adaptation mechanisms. Parameters τ, *C*, and $\bar{v}$ were determined through parameter fitting as described in section *Methods: Data analysis: parameter fitting*.

### Experimental set-up of spiking-sensor model

**Figure 3:**
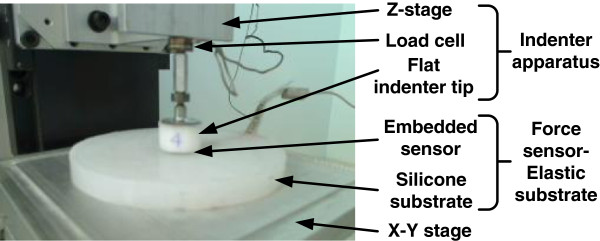
**Set-up of indenter apparatus and force sensor-elastic substrate.** The force sensor mimics the SAI mechanoreceptor inside the skin, while the load cell measures applied force at the indenter tip.

A custom-built mechanical indenter and contactor tip were used to deliver precise ramp-and-hold stimuli in the z–direction to the force sensor-elastic substrate (Figure 3).

**Figure 4:**
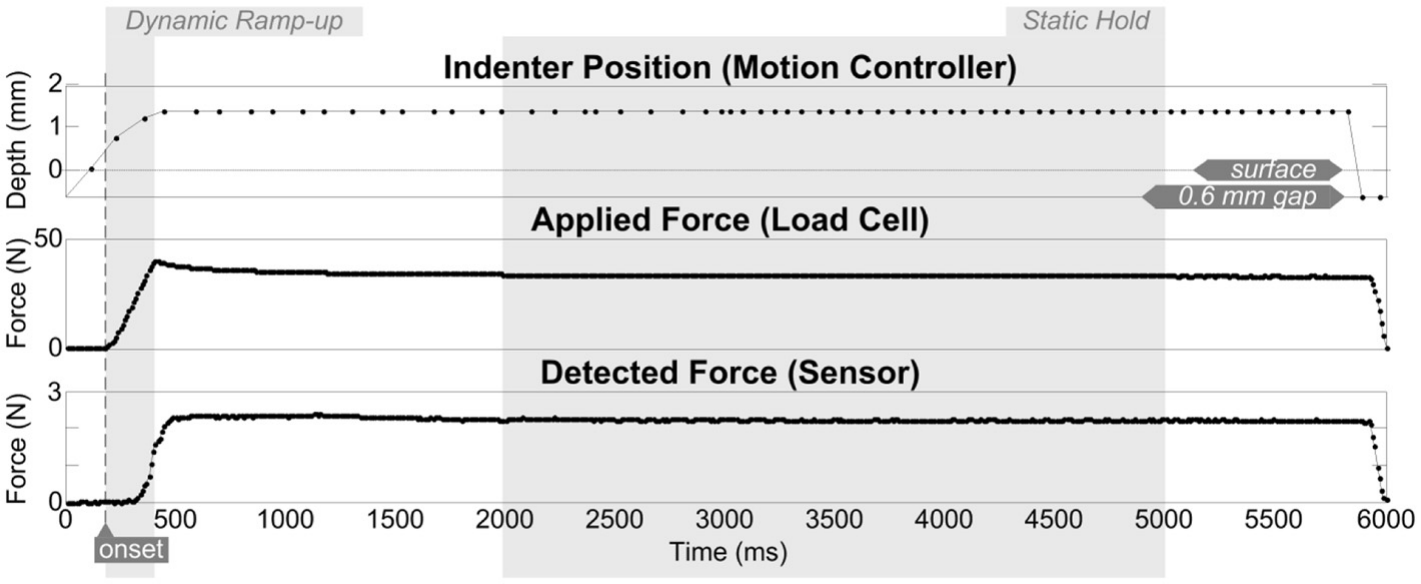
**Data collected during a ~5 s ramp-and-hold indentation.** Indentation was provided via a flat indenter tip, 1.4 mm into the surface of the sensor-substrate. Indenter tip starts from an initial position of 0.6 mm above the elastic-substrate’s surface. Phases of dynamic ramp-up and static hold are highlighted in gray.

A motion controller (ESP300; Newport Corporation, Irvine, CA) commanded a high-precision, mechanical linear z-stage (ILS100CC; maximum velocity = 100 mm/s; Newport Corporation) and reported the stage's position (resolution = 0.0001 mm), as shown in the top panel of Figure 4.

Applied force was also monitored using a low-profile load cell (Sensotec 11 subminiature; maximum load = 44 N; Honeywell Inc., Columbus, OH). Although the load cell typically outputs voltage, it was calibrated by applying small indentations to a mass scale to derive normal force (resolution = 10^-3^ N), as shown in the middle panel of Figure 4. The bottom panel of Figure 4 illustrates an example of the force sensor's response to the applied indentation as a filtered continuously-detected trace with a sampling rate of 100 Hz.

The cylindrical Delrin contactor tip, threaded to the bottom of the load cell, was oriented so that its flat end provided a rigid contact against the surface of the elastic substrate (Figure 2). The cylindrical tip’s dimensions were 14 mm height by 20 mm diameter. The use of this particular tip size and shape ensured that an equal state of normal stress was applied over the entire sensing area surface of the embedded sensor and minimized the out-of-plane shear stress [29]. These conditions thereby matched those for the mouse SAI afferent.

Directly below the indenter tip, a manually-controlled and low-profile x–y stage (443 dual-mounted; Newport Corporation) supported a square aluminum tray, on which the force sensor-elastic substrate was placed and positioned in the local horizontal plane during the experiments (Figure 3).

### Experimental set-up of mouse SAI afferent

**Figure 5:**
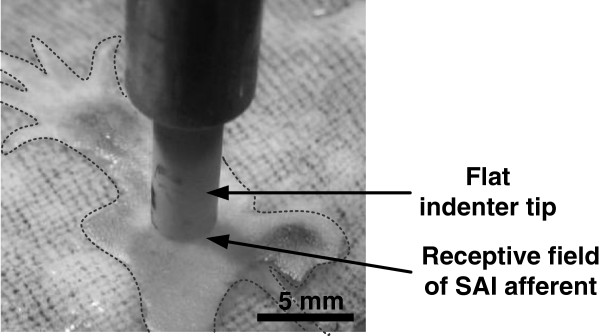
**Set-up of SAI recordings.** in mouse hairy skin (outlined) and indenter. Indenter tip is positioned over receptive field of mouse hairy skin (outlined).

A mechanical indenter and contactor tip was used to deliver vertical ramp-and-hold stimuli to the receptive field of an SAI afferent in an *ex vivo* skin-nerve preparation from a single adult female mouse (Figure 5). Thirty-eight stimulations were delivered over several hours. The first three preconditioning stimuli were omitted from analysis. Details of the experimental procedure are described in the *Methods: Common Experimental Procedure* section. All animal use was conducted according to the *National Institutes of Health Guide for the Care and Use of Laboratory Animals* and was approved by the Institutional Animal Care and Use Committee of Baylor College of Medicine and the Department of Defense.

Single SAI afferents were isolated and identified as described in Wellnitz, Lesniak, Gerling, and Lumpkin [22], summarized here. Both a portion of nerve and the surrounding hairy skin of the mouse's hind paw were dissected and pinned to a 5 mm thick silicone-elastomer substrate within a custom recording and perfusion chamber. Synthetic interstitial fluid was perfused beneath the skin while individual nerve fibers were separated and draped over an electrode for differential recording. A calibrated force fiber was then used to locate the receptive fields of single afferents and estimate their mechanical thresholds. Responses were classified as SAI afferents based on conduction velocity (>9 m/s), mechanical threshold (<1 mN), punctate receptive field (diameter <0.5 mm), slow adaptation, irregular firing pattern, low spontaneous firing rate, and directional insensitivity to stretch. SAI receptor identity was confirmed by fluorescent detection of Merkel cells in the receptive field [21].

Over the entire receptive field of the identified afferent, a linear actuator (D-A.25AB-HT17-2-BR/4; Ultra Motion, Cutchogue, NY) commanded a flat-cylindrical MACOR contactor tip (diameter = 3.42 mm; edge round radius = 0.32 mm) to provide calibrated axial displacements. A load cell (Sensotec 31 subminiature; Honeywell Inc.) was mounted to the contactor tip to monitor reaction force in Newtons. Due to noise present in the load cell’s force readings during movements with high acceleration, a low-band pass Gaussian filter, a combination of a fast Fourier transform algorithm and its inverse, eliminated all frequencies greater than 15 Hz to reconstruct cleaned force readings.

During displacements, extracellular action potentials were recorded through a differential amplifier (1800; A-M Systems Inc., Sequim, WA) and an A/D card (DT304; Data Translation Inc., Marlboro, MA) with supporting software (SciWorks Experimenter 6.0; DataWave Technologies, Loveland, CO). Spikes were collected from single afferents isolated using Sciworks [22].

### Common experimental procedure

Given both set-ups, a procedure with a randomized complete block design was performed on the respective experimental platforms, where treatments of ramp-and-hold indentations (coded as five unique indentation types) were blocked by replication. Each indentation type was defined by a unique combination of the rate of change of indentation during the ramp-up phase (3 levels: types *I*, *II*, &*III*) and the magnitude of indentation during the hold phase (3 levels: types *III*, *IV*, &*V*).

**Figure 6:**
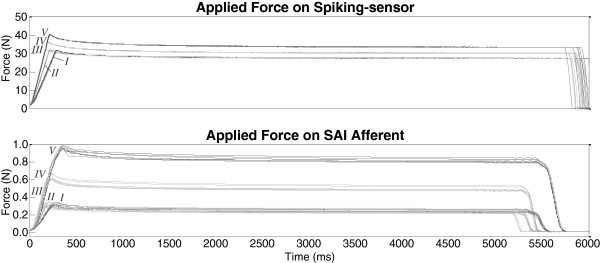
**Applied force profiles.** All replications of applied force profile traces across five types of indentation (I, II, III, IV, & V) overlaid for the spiking-sensor (top panel) and a mouse SAI afferent (bottom panel) from onset to offset. Both ramp-up rate and hold magnitude were independently varied as indicated by different shapes of the indentation types.

**Figure 7:**
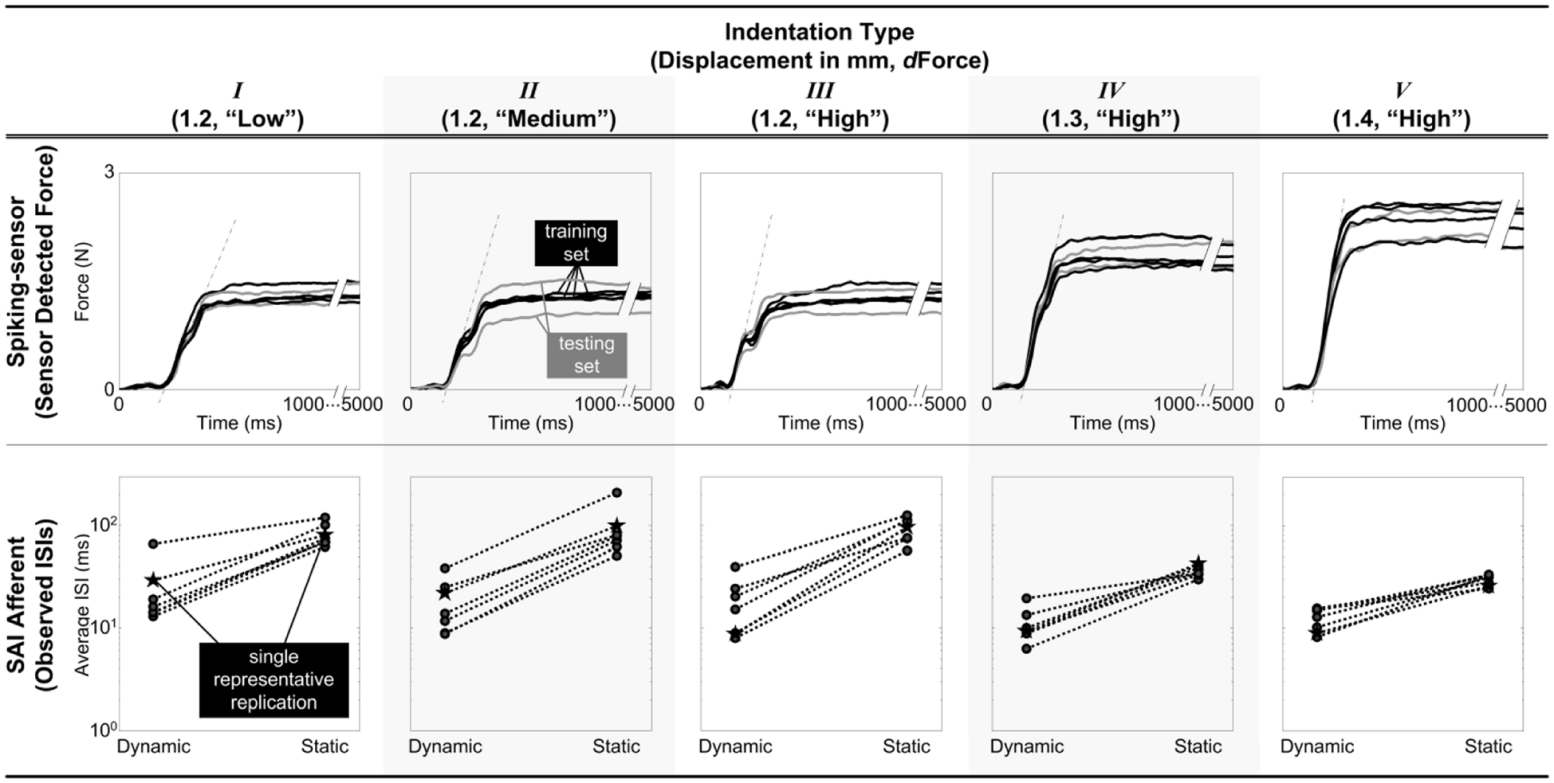
**Dependent metrics as inputs into parameter fitting and model validation procedures.** Top panel: amongst 30 stimulations across all five types, 20 were selected as training set (black) to fit the model, while the remaining 10 were reserved for the test set (gray) to validate the model. Bottom panel: amongst 35 stimulations spanning all five types, 5 (star pairs) were pseudorandomly selected to represent each of the types.

Indentation types *I*, *II*, and *III* thereby shared the same commanded magnitude of force in the sustained hold portion via a constant 1.2 mm displacement, but delivered increasing levels of ramp-up rate of change – as shown qualitatively in Figure 6 through increases in achieved ramp-up slope and quantitatively through increases in achieved maximum rate of force change, *dForce*. Although the stimuli were displacement-controlled, similar force levels were maintained between 1000 and 5000 ms of indentation (Figure 7).

Indentation types *IV* and *V* shared the same ramp-up rate of change as *III*, but delivered increasing magnitudes of force in the sustained hold portion via 1.3 and 1.4 mm displacements – shown qualitatively in Figure 6 through increases in achieved plateau height, but with similar ramp-up slope. Thirty ramp-and-hold indentations were presented to the force sensor-elastic substrate (five indentation types × six replication blocks), while 35 were presented to the mouse SAI afferent (five types × seven blocks).

Following this experimental design, the indenter tip was centered and positioned above each respective experimental platform's receptive field before conducting the replication blocks. Within each replication block, all five types were performed in random order. All indentations shared the same starting position of 0.6 mm above the experimental platform’s surface, as described in Wellnitz, et al. [22]. Additionally, all indentations consisted of a ramp-up phase followed by a sustained ~5 s hold phase and were presented at 60 s intervals. Periods of no stimulation reduced stimulus adaptation and allowed sufficient time for the experimenter to control the indenter.

Prior to running each experiment, platforms were preconditioned. During a preconditioning session, the indenter tip – at a velocity of 6 to 8 mm/s – applied three indentations to a depth of 1.2 mm into the experimental platform from a starting gap of 0.6 mm above the platform’s surface. Preconditioning indentations were not included in the analysis. In the preconditioning process, it was evident that after three indentations, the force recorded at the probe tip was consistent between subsequent indentations of equal depth, as had been considered elsewhere [30]. Over the first three indentations, the force recorded while the probe was held at its commanded depth decreased significantly between trials, due to initial relaxation of the elastic substrate and skin specimens.

### Data analysis: independent and dependent metrics

We experimentally controlled for rate of ramp-up and magnitude of hold to elicit continuously detected force over time from the force sensor and spikes over time from the SAI afferent. The independent metrics are coded treatments of indentation types *I*, *II*, *III*, *IV*, and *V* and the dependent metric is average interspike interval, or $\bar{ISI}$, during separate phases of stimulus ramp-up ( $\bar{dynISI}$) and hold ( $\bar{statISI}$), in Eq. 6 – 8.

Since

(6) $$ISI_{i}=t_{i+1}-t_{i}$$

or simply the time difference between two consecutive spikes, $\bar{ISI}$ is calculated by dividing the sum of all *ISI*s within a given time window by the total number of *ISI*s contained within the same window. Thus, the dynamic $\bar{ISI}$ (7) or

(7) $$\bar{dynISI}=\frac{\sum_{i=0}^{m-1}\left(ISI_{i}\right)}{m}$$

is calculated by dividing the sum of all *ISI*s from the time of stimulus onset to the time of peak indentation by the total number of *ISI*s, m, within the same window. Stimulus onset, for the spiking-sensor model, was defined as when the magnitude of applied force from the load cell exceeded 1 N. Stimulus onset, for the mouse SAI, was defined as when *dForce* from the load cell exceeded 0.5 mN/ms. Time of peak indentation for both set-ups was defined as when magnitude of applied force reached maximum. The static $\bar{ISI}$ (8) or

(8) $$\bar{statISI}=\frac{\sum_{i=n}^{p-1}\left(ISI_{i}\right)}{p-n}$$

is calculated by dividing the sum of all *ISI*s from 2 to 5 seconds by the number of those *ISI*s, *p - n*. This selected window incorporates the later portion of typical double exponential adaptation of an SAI afferent [31]. Thus, *t*_*n*_ is the time of spike upon entering static hold phase, while *t*_*p*_ is the spike time before ending static hold phase. Data were analyzed using *ISI* of the time domain, since this requires less transformation of the data through our model than a measure such as firing rate in the frequency domain. In addition, we preliminarily examined first spike latency in ms.

Figure 7 illustrates the dependent metrics used as input into parameter fitting and model validation procedures. From the experiment with the spiking-sensor model, the 30 traces of sensor-detected force were randomly split amongst a training set (two-thirds of the total data set: 20 traces) and test set (remaining one-third: 10 traces). The training set was used for parameter fitting while the test set was reserved for model validation. In the experiment with the mouse SAI afferent, the 35 calculations each of $\bar{dynISI}$ and $\bar{statISI}$ were made (five indentation types with seven replications), from which we selected five $\bar{obsdynISI}$ and five $\bar{obsstatISI}$ to represent a typical SAI afferent’s response respectively to the dynamic ramp-up and static hold, given each unique application of an indentation type (Figure 7, bottom panel).

### Data analysis: parameter fitting

The spiking-sensor model’s neural firing predictions for dynamic ramp-up and static hold phases were fit to those observed in the mouse SAI afferent. The parameters of the mathematical neuronal spiking model were fit using a training set, before validating via test set the fitted model's spike time predictions against those observed in the SAI afferent. Two parameter fitting sessions were performed with a different set of parameter values.

The parameters were fit using response surface methodology (RSM). The general RSM process is typically driven by the goal of optimizing a response, which is influenced by one or many factors. This work strives to maximize the fractional sum of squares (*FSS*), previously used by Phillips and Johnson (1981) [12], between predicted and observed spike times, which is influenced by the six model parameters: *ß*, *k*_*s*_, *k*_*d*_, τ, *C*, and $\bar{v}$. Specifically, *FSS* is a complement of normalized sum of squared deviations and is contributed equally by dynamic (*ω*_*dyn*_ = 0.5) and static (*ω*_*stat*_ = 0.5) comparisons of spiking response (9).

(9) $$\begin{array}{l} FSS=\omega_{\mathrm{dyn}}\left[1-\frac{\sum_{i=1}^{5}\sum_{j=1}^{4}{\left(\bar{obsdynISI_{i}}-\bar{preddynISI_{\mathrm{ij}}}\right)}^{2}}{4\sum_{i=1}^{5}{\left(\bar{obsdynISI_{i}}\right)}^{2}}\right]\\ \;+\omega_{\mathrm{stat}}\left[1-\frac{\sum_{i=1}^{5}\sum_{j=1}^{4}{\left(\bar{obsstatISI_{i}}-\bar{predstatISI_{\mathrm{ij}}}\right)}^{2}}{4\sum_{i=1}^{5}{\left(\bar{obsstatISI_{i}}\right)}^{2}}\right] \end{array}$$

The terms $\bar{preddynISI_{\mathrm{ij}}}$ and $\bar{predstatISI_{\mathrm{ij}}}$ are the $\bar{dynISI}$ and $\bar{statISI}$ pairs predicted by the spiking-sensor during replication *j* (amongst the 20 traces in the training set) in response to indentation type *i* (indentation type conditions *I*–*V*) using a specified set of model parameter values.

### Data analysis: model validation

The final FSS achieved in parameter fitting was compared to an FSS calculated using the two stimulations reserved for the test set, using the fitting session’s final set of parameters. Although a high *FSS* was desired, the test set *FSS* was hypothesized to be lower than that calculated using the training set, since the alternative would indicate overfitting the model.

Next, the effect of the rate during dynamic ramp-up and the magnitude during static hold on predicted $\bar{ISI}$ were analyzed. Since both experiments presented to the spiking-sensor model and SAI afferent were of randomized complete block design, the general treatment effect of indentation type on both $\bar{dynISI}$ and $\bar{statISI}$ was analyzed before further pair-wise comparisons were made. An analysis of variance (ANOVA) was conducted to test the null hypothesis of no treatment effect on $\bar{dynISI}$ and $\bar{statISI}$. ANOVA was conducted on both the observed and predicted spike firing responses. Although the five indentation types were blocked by the order of experimental blocks, only the treatment effect was tested. The blocking effect on spiking response addresses the adaptation of the spiking-sensor model and SAI afferent to repeated stimuli. Although an intriguing topic, it is not within the scope of this work. As a follow-up post hoc analysis, a Tukey’s test was conducted for multiple pair-wise comparisons to test the individual null hypotheses of equal treatment means. All statistical analyses were conducted at a 0.05 significance level.

## Results

### Parameter fitting results

Table 1 focuses on the second session and provides the values of the six model parameters (*ß*, *k*_*s*_, *k*_*d*_, τ, *C*, and $\bar{v}$) and their respective calculated *FSS* for each of the iteration steps. We demonstrated that RSM is sensitive to starting conditions by the different paths taken by the two fitting sessions. The first session ended with a parameter set that produced a worse fit (*FSS* = 0.829 with parameter values 4.33E-08 mA, 5.74E-07 mA/N, 1.01E-03 mA· s/N, 71.592 ms, 1.01E-06 mF, 50.723 mV) than that produced by the second session’s set of parameters (*FSS* = 0.936 with parameter values 2.72E-08 mA, 6.20E-07 mA/N, 2.71E-04 mA· s/N, 71.409 ms, 9.70E-07 mF, 47.300 mV).

**Table 1:** Parameter fitting session

**Iteration**	**Model parameters**						
	***ß*(mA)**	***k***_***s***_**(mA/N)**	***k***_***d***_**(mA· s/N)**	***τ*(ms)**	***C*(mF)**	$\bar{\nu }$**(mV)**	***FSS***
0 (Start)	0	6.00E-07	2.60E-04	68.000	1.00E-06	50.000	0.748
1	1.82E-08	6.23E-07	2.74E-04	70.409	9.78E-07	47.000	0.935
2 (End)	2.72E-08	6.20E-07	2.71E-04	71.409	9.70E-07	47.300	0.936

**Figure 8:**
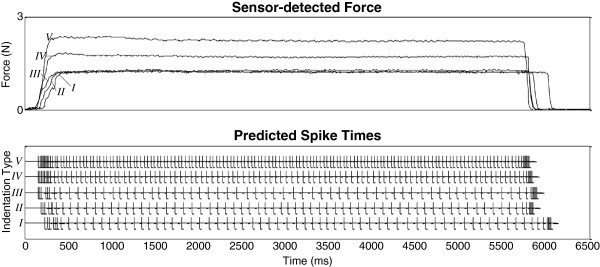
**Data transformations using final parameter values.** Using model parameter values obtained from final fitting iteration of second parameter fitting session, sensor-detected force is transformed to predicted spike times.

All following analyses focus on using the parameter values obtained during the second parameter fitting session. Using the final model parameter values obtained in the last iteration step of the second parameter fitting session, Figure 8 portrays single translations of sensor-detected force into trains of spike times across indentation types. These qualitative results demonstrate increased spike density in the static phase as the indentation’s magnitude increases. In addition, increased spike density and earlier first spike latency can be seen in the dynamic phase as the indentation’s ramp-up rate of change increases.

### Model validation results

*FSS* calculated from both the training and test set were compared. Comparing the predicted spiking response to those observed upon reaching the end of parameter fitting resulted in a *FSS* of 0.936, which consisted of dynamic ramp-up and static hold components, 0.904 and 0.968 respectively. In comparison, the differences between predicted and observed spiking response when using the test set resulted in a lower *FSS* of 0.888, where the dynamic ramp-up and static hold components were 0.895 and 0.881 respectively. This decrease in performance does not suggest a poor fit since the test *FSS* was still greater than 0.8. Instead, it suggests the model was not overfit.

Next, the general treatment effects of indentation type on $\bar{dynISI}$ and $\bar{statISI}$ were tested on both predicted and observed spiking response. At a significance level of 0.05, the null hypothesis, that indentation type has no effect on predicted and observed $\bar{dynISI}$, is rejected (F_NullPredicted_ = 16.45 > F_0.05,4,20_ = 2.87; F_NullObserved_ = 3.86 > F_0.05,4,24_ = 2.78); thus, the $\bar{dynISI}$ means differ when both predicted by the spiking-sensor model (p-value < 0.01) and observed in the SAI afferent (p-value < 0.025). At a significance level of 0.05, the null hypothesis (H_Null_ = indentation type has no effect on predicted and observed $\bar{statISI}$) is rejected (F_NullPredicted_ = 16.52 > 2.87; F_NullObserved_ = 11.49 > 2.78); thus, the $\bar{statISI}$ means differ when both predicted by the spiking-sensor (p-value < 0.01) and observed in the SAI (p-value < 0.01).

**Figure 9:**
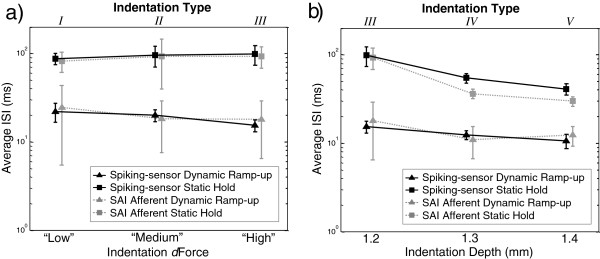
**Average*ISI*compared between spiking-sensor model and SAI afferent spike times.** Across **a)** increasing indentation ramp-up dForce and **b)** increasing indentation depth, average *ISI* (error bars = SD) is compared between spiking-sensor model and SAI afferent spike times for dynamic ramp-up and static hold phases. Note log scale used on y-axes.

Across increasing stimulus ramp-up rates, Figure 9a compares the mean $\bar{dynISI}$ and $\bar{statISI}$ (±SD) between predicted and observed responses. The Tukey method’s multiple pair-wise comparisons of $\bar{dynISI}$ and $\bar{statISI}$ means across the indentation types *I*, *II*, and *III* (increasing ramp-up rate from low, medium, to high, respectively) suggest that at a significance level of 0.05, only the *I* vs. *III* type pairs of $\bar{dynISI}$ means significantly differed (difference > 5.01 ms, given q_0.05, 5, 20_ = 4.24) for the spiking-sensor. None of the pairs of $\bar{dynISI}$ means observed in the SAI afferent were significantly different (all differences < 11.53 ms, given q_0.05, 5, 24_ = 4.17). For both the spiking-sensor and SAI afferent, all type pairs *I* vs. *II*, *I* vs. *III*, and *II* vs. *III* of $\bar{statISI}$ means were not significantly different (all differences < 27.19 and < 38.50 ms, respectively).

Across increasing stimulus hold magnitudes, Figure 9b compares the $\bar{dynISI}$ and $\bar{statISI}$ (± SD) between predicted and observed responses. The Tukey method’s multiple pair-wise comparisons of $\bar{dynISI}$ and $\bar{statISI}$ means across the indentation types *III*, *IV*, and *V* (increasing hold magnitude from 1.2, 1.3, to 1.4 mm, respectively) suggest that at a significance level of 0.05, none of the pairs of $\bar{dynISI}$means significantly differed for those predicted by the spiking-sensor (all differences < 5.01 ms, given q_0.05, 5, 20_ = 4.24) and those observed in the SAI afferent (all differences < 11.53 ms, given q_0.05, 5, 24_ = 4.17). For both spiking-sensor and SAI afferent, the pairs of $\bar{statISI}$ means from *III* vs. *IV* and *III* vs. *V* were significantly different (differences > 27.19 and > 38.50 ms, respectively).

### First spike latency results

As rate of stimulus ramp-up increased, mean first spike latencies (± SD) decreased for those predicted by the spiking-sensor (244.90 ± 8.81, 192.28 ± 6.19, 154.96 ± 3.17 ms). As magnitude of stimulus hold increased, for indentation types *III*, *IV*, and *V* (1.2, 1.3, 1.4 mm respectively), mean first spike latencies (± SD) were not different for those predicted by the spiking-sensor (154.96 ± 3.17, 155.525 ± 4.55, 159.055 ± 5.86 ms) although they exhibited an increasing trend. These predictions are an order of magnitude greater than first spike latencies recorded from the mouse SAI afferent, which exhibited a range from 0.81 to 96.92 ms across all indentation types, a point considered in the *Discussion* section.

### Interspike interval irregularity results

In addition, we qualitatively compared the effect of low-pass filtering out frequencies greater than 15 Hz on the production of irregular *ISI*s. For instance, when spike firing was predicted from raw force sensor data, a coefficient of variation (CV) for static *ISI* of 0.16 was reported. When the same raw force sensor data trace was low-pass filtered (frequencies > 15 Hz), the predicted CV for static *ISI* decreased to 0.07. Under similar stimulation, the mouse SAI afferent reports mean CV value of 0.78 ± 0.09 for static *ISI*. This suggests that unfiltered data from the force sensor aids in the generation of irregular *ISI*s, though the resultant CV value is still not as great as that observed in the SAI afferent case.

## Discussion

This effort takes an integrated engineering approach to artificially replicate the neural firing behavior of an SAI afferent in its response to both magnitude and rate of indentation force by integrating a physical force sensor, embedded in an elastic substrate, with mathematical models of membrane transduction and neural dynamics. We sought to mimic particular SAI neural firing features which include increased response to changes in stimulus magnitude and rate of change, as well as the SAI afferent’s steady response to sustained stimuli and differentially greater response to moving stimuli. These responses to ramp-and-hold, sustained stimuli tie to tasks of contacting and holding an object. Importantly, the input which drives the models was the force recorded within the elastic substrate, of distinct form and delay between force or displacement at the tip of the probe and the surface of the sensor.

The effort is unique because it begins to account for skin elasticity by measuring force from within simulated skin, utilizes only six free parameters, and separates parameter fitting and model validation using response surface methodology. It also focuses upon the paradigm of the ramp-and-hold stimulus rather than vibration. By addressing these gaps, the effort extends prior works. First, prior efforts to predict the characteristic features within trains of action potentials have not taken into account the mechanics of the skin in the propagation of forces through elastic skin substrates toward the locations of the natural end organs, but rather use as input the position of the stimulus (as well as velocity, acceleration and jerk) relative to the surface contact or reaction force at the stimulus tip [6]. However, it is clear from many past skin mechanics studies that the skin’s structure significantly influences and reshapes this propagation of forces. The forces at the tip of the indenting object are quite different from the forces at the site of end organ mechanotransduction. Moreover, particular quantities of stress and/or strain near the locations of end organs (e.g., max compressive strain and strain energy density) have been correlated to spike firing rate during the stimulus hold phase. Though the exact quantity remains an elusive question, no practical sensor can sense complex stress and strain properties, nor be robust enough to be embedded within an elastic skin-like substrate. Therefore, while work here is a start, further efforts are needed to understand and account for the many complexities of the skin (e.g., exact stress/strain quantity, rheological and tribiological characteristics, undulating ridge structure [16,32], etc.). Second, prior efforts focused upon vibratory stimuli, whereas this work sought to mimic the elicitation of trains of action potentials, in particular, those essential features captured by the slowly adapting type I (SAI) afferent in response to everyday tasks of contacting and holding an object. Cues used here in the differentiation of various stimulus pressures, are of great importance for prosthetics users who want to hold a child’s hand or grasp a glass (i.e., “on-off” or “how much”) and match with the SAI afferent’s characteristic response to held indentation with sustained firing, and to increased hold magnitude and rate of indentation with linear increases in firing rate. The ramp-and-hold paradigm is also central to important and long-standing questions regarding SAI adaptation, first spike timing, and discrimination of displacement depth and force. Finally, this work utilizes and fits six free parameters in comparison to the 100–150 used in other works [6].

The model predictions fit well with observations of the characteristic features within trains of SAI action potentials elicited in the mouse. In specific, the computational model replicated basic findings regarding the mouse SAI afferent’s discrimination of levels of stimulus magnitude in the static hold phase. As the magnitude of indentation increased from 1.2 mm (indentation type *III*), 1.3 mm (*IV*), to 1.4 mm (*V*) the spiking-sensor model predicted a trend of increased spike firing, evident from decreasing average *ISI*s (98.81, 54.51, 41.11 ms respectively) within the static hold phase (Figure 8b). These predictions fit favorably (mean *stat_FSS* = 0.925) to those observed average static *ISI*s (93.31, 36.28, 29.89 ms respectively) and agree with works by Mountcastle et al. [33] and Ge and Khalsa [29]. Mountcastle et al. [33] demonstrated a roughly positive and linear relationship between stimulus displacement magnitude and firing rate reported by SAI afferents in the macaque monkey. An increase in magnitude from 0.2 to 0.9 mm related to an increase from an average of 0 to 70 impulses/s, or a decrease in average *ISI*s from 1000 to 4.29 ms. Ge and Khalsa’s work [29] demonstrated that an increase in indentation magnitude from 0.05 to 0.2 mm related to a linear increase (R^2^ = 0.72) in firing rate response from 0 to 20 impulses/s, or a decrease in average *ISI*s from 1000 to 50 ms. While the findings from both works did not share the exact magnitude of stimuli, the trend of response to increasing magnitude is consistent. Inconsistencies in response range may be due to, but not limited to, different tactile sensitivities across animal species.

Furthermore, this work replicated the mouse SAI afferent’s discrimination of rates of stimulus ramp-up. Regarding the fit of predicted and observed *ISI*s in the dynamic ramp-up phase, as the rate of indentation increased during the ramp-up phase from 1.43 N/ms (indentation type *I*), 1.78 N/ms (*II*), to 2.19 N/ms (*III*), the spiking-sensor model predicted a trend of increased spike firing, inferred from decreasing average *ISI*s (21.85, 19.98, 15.42 ms respectively) in the dynamic ramp-up phase (Figure 8a). These predictions fit favorably (mean *dyn_FSS* = 0.900) to those observed average dynamic ramp-up *ISI*s (24.58, 18.32, 17.86 ms respectively). The closest connection to these findings is work by Pawluk [34] in which ramp-up stimuli were used. However, that work did not provide quantitative descriptions, with which one could directly compare. Indirectly related to the rates of stimulus ramp-up used here are vibration studies, if one considers that increased frequency corresponds indirectly. In those studies employing repeated sinusoidal vibration, increases in frequency from 10 to 60 Hz lead evoke increases the SAI afferent response from ~23 to 42 impulses/s (or a decrease in $\bar{ISI}$s from 43.48 to 23.81 ms) in a macaque monkey [35].

### First spike latency

In addition to *ISI*, others have sought to tie the timing of the first few neural spikes to perception. We have preliminarily analyzed the time elapsed between stimulus onset and the first spike elicited, or first spike latency, as stimulus magnitude and ramp-up rate are changed. Our predicted first spike latencies exhibit decreasing trends as both ramp-up rate and hold magnitude was increased. In addition, the predictions are an order of magnitude greater than both those recorded from the mouse SAI afferent and reported by Johannson [36] for human SAI afferents (range of ~20 to 90 ms).

We believe the discrepancy to be due to our model fitting procedure’s exclusion of this metric, filtering artifacts, responsiveness of the sensor in silicone (see section *Responsiveness of sensor and load cell*), and sensor location and size. Furthermore, current force sensors may not respond on the timescales of biological mechanoreceptors, which exceeds even our analog load cell. Future efforts will seek to better match first detection of stimuli to biological scales through improvements in modeling and sensor miniaturization.

### Interspike interval irregularity

In addition to the presented findings, future efforts might recreate the irregularity of neuronal spike firing. Although our model predicts regular *ISIs*, it may be useful to match to the highly-variable *ISI*s that are characteristic of SAI responses [22,37]. Noise in the nervous system may originate from innate signal transduction mechanisms, or synaptic transmission, might be a consequence of SAI neurite arbor integration, or may stem from other unknown sources. It may be simulated using a combination of or one of two methods: adding noise or manipulating sensor-output filtering. The first method could simulate the introduction of noise directly into the computation model, as demonstrated by Russell et al. [38], where Gaussian noise is added after the filtering processes. The second method could reconsider our filtering procedure of the sensor analog voltage output and reintroduce a small band of frequencies and thereby a small level of variance in computations of both current and membrane potential.

### Responsiveness of sensor and load cell

**Figure 10:**
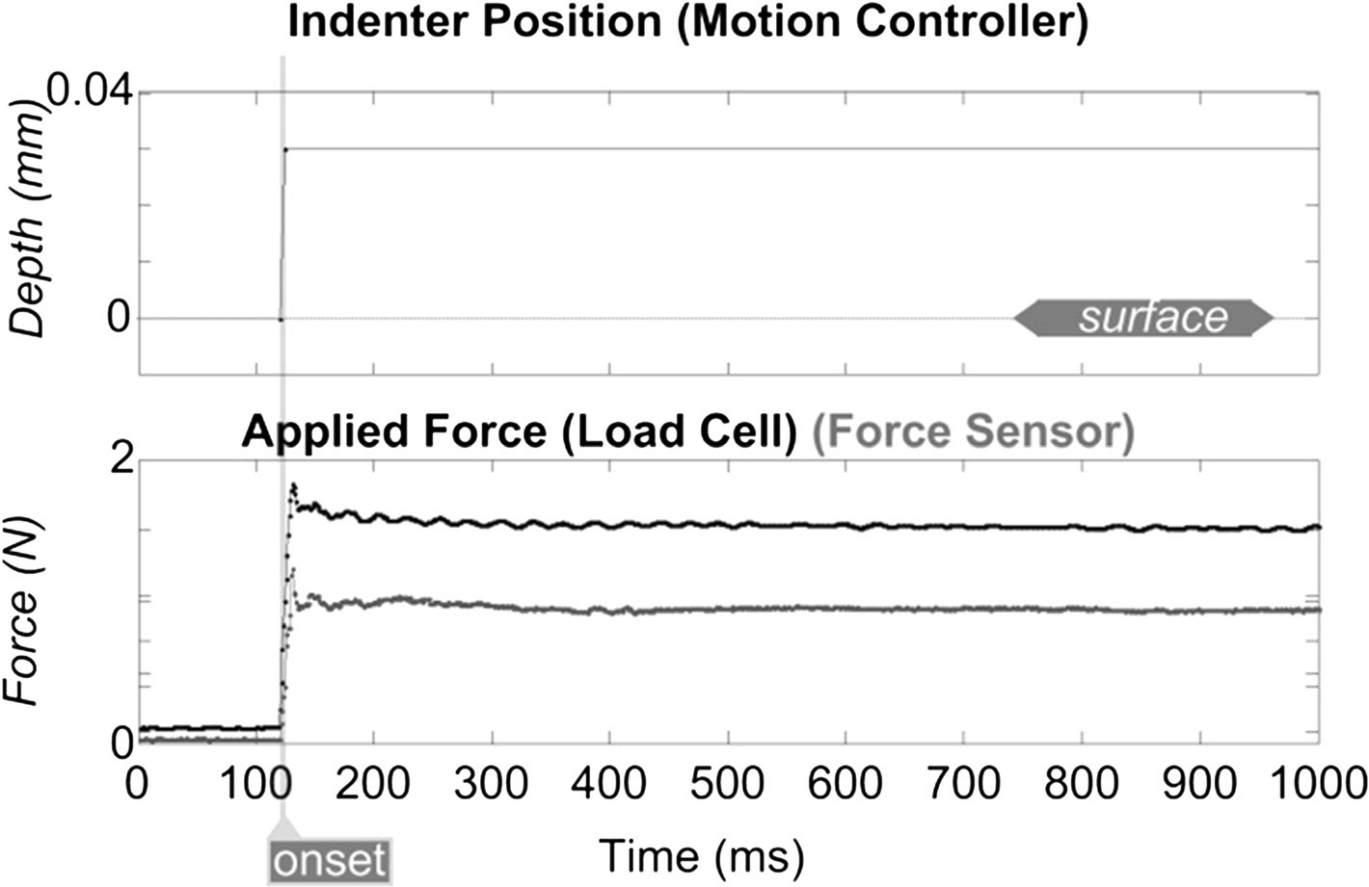
**Responsiveness of sensor and load cell.** 0.4 mm indentation of the probe tip (top) into a Flexiforce sensor separated from the elastic substrate (bottom, gray trace) is plotted with the response from the load cell (black trace). The slight initial offset of the load cell trace helped differentiate the curves.

The onset response of the Flexiforce sensor, apart from the silicone-elastomer substrate was also analyzed. As is observable in Figure 10, the response of the load cell’s trace lags the displacement trace slightly, as does that of the Flexiforce sensor. However, the onset timing of the responses of Flexiforce sensor and load cell are identical. This indicates that the temporal lag in sensor response (Figure 4, lower trace) is predominantly due to its encasement in an elastic substrate, analogous to the skin’s effects on mechanoreceptor response in the biology [39].

### Direct nerve interfacing

Finally, toward the goal of direct nerve interfacing, we note that work has been done to deliver trains of current pulses by directly inserting electrodes into sensory fibers of the median nerve [40]. Current pulses depolarized nerves to elicit action potentials. Some of these researchers validated action potential elicitation against expected signal trains while others recorded psychophysical graded perceptions. At present, the majority of their work has focused upon the transformation of signal from force sensor to current pulse not informed by native transduction mechanisms. While our work herein has sought to generate the characteristic features within trains of action potentials, the next steps are to link this model with current pulse waveforms that stimulate single action potentials at the peripheral afferent.

## Competing interests

All authors declare that they have no competing interests.

## Authors’ contributions

All authors have made substantial contributions in conception, design, data acquisition, analysis, and interpretation of data. All authors have been involved in drafting the manuscript and revising it critically. All authors have read and approved of the final manuscript.

## Authors’ information

Elmer K. Kim is a research engineer in the University of Virginia's Department of Systems and Information Engineering. His research interests focus on human factors and human-computer interaction. Currently, he is developing technologies for sensory neural prosthetics. He earned his B.S and M.S. degrees in Systems Engineering from University of Virginia.

Gregory J. Gerling received his Ph.D. degree from the Department of Mechanical and Industrial Engineering, the University of Iowa, Iowa City. He is an associate professor in the Department of Systems and Information Engineering, University of Virginia, Charlottesville. His research interests include haptics, computational neuroscience, human factors/ergonomics, and human–computer interaction.

Sarah M. Bourdon is a graduate student in the Department of Neuroscience at Baylor College of Medicine. She worked as a research technician also at Baylor College of Medicine. During this time, she studied how the sense of touch is encoded. As a graduate student, she now focuses on neurodegeneration, in hopes of understanding diseases such as Alzheimer’s.

Scott A. Wellnitz and Kim, Wellnitz, Bourdon, Lumpkin, Gerling received his Ph.D. in 2010 from the Department of Neuroscience at Baylor College of Medicine. He is currently a postdoctoral associate at the J. David Gladstone Institute for Neurological Disease. His current work focuses on molecular mechanisms of Alzheimer's disease pathology.

Ellen A. Lumpkin performed her Ph.D. research in sensory neuroscience with Dr. A. James Hudspeth at UT Southwestern Medical Center and The Rockefeller University. Her research program aims to define molecular and cellular mechanisms of cutaneous touch sensation in mammals. She is an Associate Professor of Dermatology and of Physiology and Cellular Biophysics at Columbia University College of Physicians and Surgeons.
